# Application of gamma irradiation on morphological, biochemical, and molecular aspects of wheat (*Triticum aestivum* L.) under different seed moisture contents

**DOI:** 10.1038/s41598-022-14949-6

**Published:** 2022-06-30

**Authors:** Davood Kiani, Azam Borzouei, Sanaz Ramezanpour, Hasan Soltanloo, Safoora Saadati

**Affiliations:** 1 Crop and Horticultural Science Research Department, Bushehr Agricultural and Natural Resources Research and Education Center, AREEO, Bushehr, Iran; 2 Agriculture Research School, Nuclear Science and Technology Research Institute, PO Box 31485-498, Karaj, Iran; 3 Plant Breeding and Biotechnology Department, Gorgan University of Agricultural Sciences and Natural Resources, PO Box 4918943464, Gorgan, Iran

**Keywords:** Plant breeding, Plant physiology

## Abstract

Nuclear technology is currently used as a tool in mutation breeding to improve crops by increasing genetic variation. The ionization of gamma rays produces large amounts of free radicals, simulating stressors in the natural environment. To avoid gamma-ray-induced oxidative stress, plants use antioxidant defense systems. Exposure of plants to irradiation can affect the germination, growth, and production of metabolites. Plants' sensitivity to irradiation depends on genetic and environmental factors such as moisture content. For this purpose, the effects of different gamma irradiation doses [0, 100, 200, 300, and 400 Gray (Gy)] and different seed moisture contents (7, 13, and 19%) on traits such as seed germination, seedling growth, molecular and biochemical alterations in antioxidant enzymes were examined in the current study. Based on the results, the highest seed germination percentage was observed in the interaction effect of seed moisture at 13% with an irradiation dose of 400 Gy (98.89%). Seedling survival percent and seedling length decreased with increasing doses of gamma irradiation at different seed moisture contents. Increasing gamma irradiation doses were reduced root and stem fresh and dry weight, and root and stem length. The highest level of catalase enzyme activity and expression was observed at 200 and 300 Gy irradiation doses at different moisture contents. The peroxidase and polyphenol oxidase gene expression were reduced at all contents of gamma irradiation doses and seed moisture compared to the control. It can be concluded that the dose of 200–300 Gy of gamma irradiation reduced plant growth by 30% in terms of fresh and dry weight and length of plants, as well as enhanced the expression of antioxidant enzymes. The results of this study could help plant breeders select an appropriate dose rate in wheat for further research.

## Introduction

The role of plant breeding in increasing food production and providing sustainable nutrition is well recognized^1^. Genetic variety is essential for both evolution and practical breeding^2^. Spontaneous mutations (naturally occurring) were used to great effect in the beginning stages of plant breeding. The most well-known instance is the use of wheat and rice semi-dwarf mutants during the green revolution. When it was discovered that ionizing irradiation might alter organisms' genetic make-up, plant breeding took a giant step forward^2^. The natural mutation rate is quite low but launched a new era for crop improvement^3^. Mutation induction is currently a well-established strategy in plant breeding that can supplement existing germplasms and improve cultivars with certain qualities as well.

Grains like wheat (*Triticium aestivum* L.) are important for meeting nutritional demands worldwide, but they're particularly important in underdeveloped countries^4^. Due to the limited genetic diversity among existing wheat genotypes, the development of superior varieties is hampered greatly. It is necessary that a gene pool increasing the plant can be protected against environmental stress^5^.

Gamma rays are short-wavelength electromagnetic irradiations with a high penetration depth that are produced when certain elements undergo radioactive disintegration. It has important effects on plant growth and development by changes of morphological, physiological, biochemical, genetic, and cytological in cells and tissues depending on the irradiance^6^. Many investigations have revealed the effects of gamma irradiation on seed germination, seedling growth, and oxidative stress^7–10^. It is generally known that high doses of gamma rays adversely affect the biochemical and physiological properties of plants^11–13^. However, radiosensitivity depends on the type of plant (differences in DNA content, cell cycle dynamics, antioxidant response, and repair process) and environmental conditions (such as seed moisture content, oxygen, and temperature)^14–16^. Moisture affects the seed's sensitivity to irradiation due to its role in respiration and gas transport, although the mechanism of this effect is not fully understood. For example, in barley seeds at rest, moisture content of less than 14% leads to increased sensitivity to X-rays and gamma rays^17^. It may be attributable to the ability of drier seeds to retain more radicals induced from irradiation. Wet seeds, on the other hand, have a higher respiration rate^15^. Therefore, it is important to establish the seed moisture content at rest and adjust it before irradiation. Before irradiation, seeds can be brought to different water contents ranging from 2 to 14% in a vacuum desiccator^18^.

In the presence of gamma irradiation, reactive oxygen species (ROS) such as superoxide anions, hydrogen peroxide, and hydroxyl radicals are generated, which potentially induced oxidative stress^19^. These radicals are highly reactive with a wide range of biological macromolecules like DNA, lipids, and proteins^20,21^. However, ROS levels rise when plants are subjected to biotic or abiotic stress conditions. A plant that successfully copes with one type of stress is likely to become better resistant to many other types of stress when exposed to multiple types of stress. This phenomenon is called cross-tolerance, and it suggests that plants have developed powerful defensive regulatory pathways that allow them to adapt rapidly to changing environments^22^. To avoid gamma-radiation-induced oxidative stress, plants use antioxidant defense systems such as catalase (CAT), peroxidase (POD), and polyphenol oxidase (PPO). Several studies have found that increasing antioxidant capacity can help avoid harm from ROS production^23,24^. H_2_O_2_ is formed as a result of superoxide dismutation, which is hazardous and necessitates detoxification by other enzymes. CAT (EC 1.11.1.6) is an enzyme that breaks down H_2_O_2_ into O_2_ and H_2_O. In the ROS-defense network, the role of catalase is unclear. Animals, plants, and microorganisms all have a POD (EC 1.11.1.7), which oxidizes an extensive range of substances (electron donors) when hydrogen peroxide (H_2_O_2_) is present^25^.

Previous studies have investigated the effect of gamma radiation dose on seed germination and biochemical and physiological changes in plants^26,27^. This study aimed to determine the optimal dose of gamma irradiation as well as the resistance to oxidative stress induced by gamma irradiation by investigating the morphological, biochemical, and molecular traits in response to different doses of gamma radiation and seed moisture content of wheat (*Triticum aestivum* L. var. Mahdavi).

## Results

### Morphological traits

**Figure 1:**
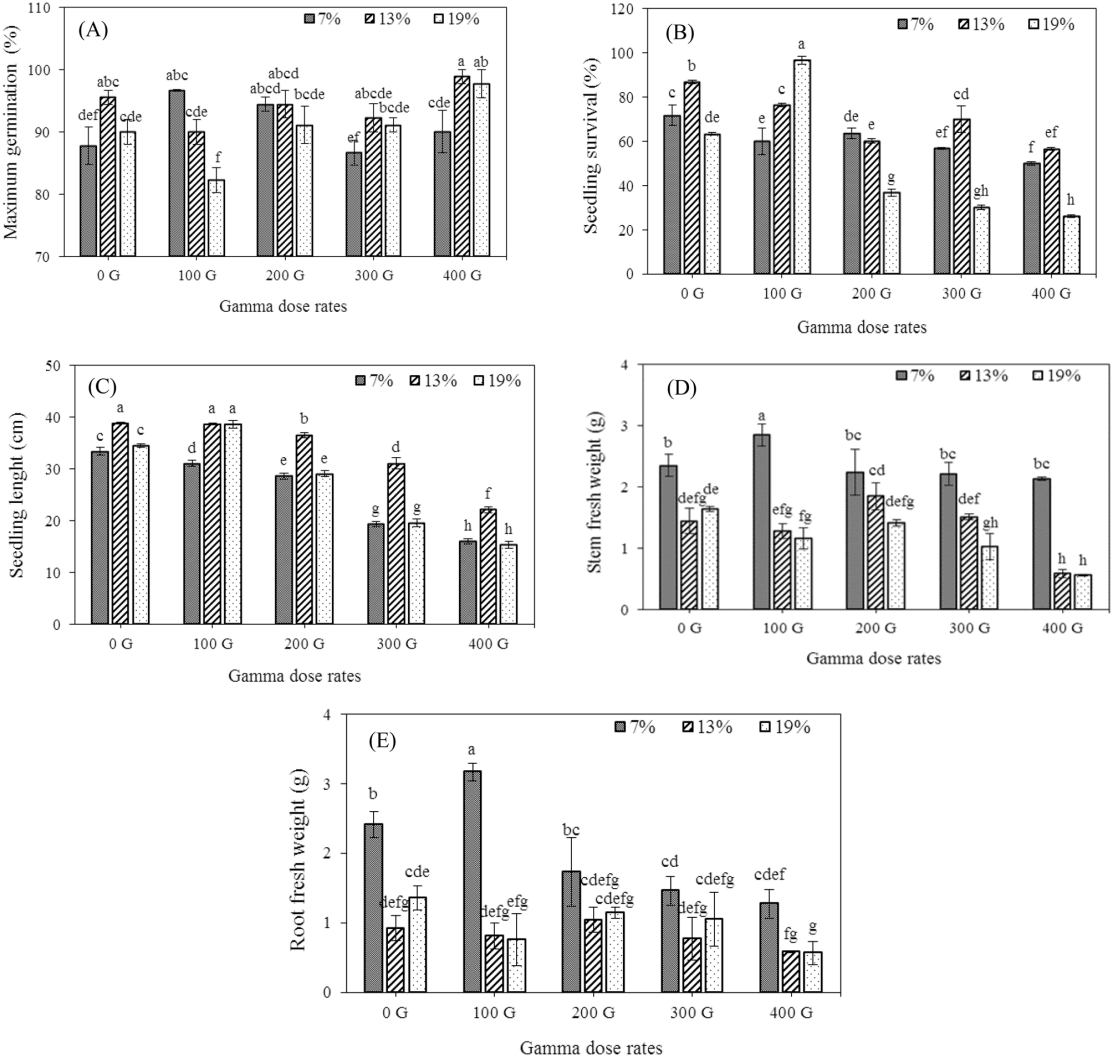
Effects of gamma dose rates and different seed moisture contents on maximum germination (**A**), seedling survival percent (**B**), seedling length (**C**), stem fresh weight (**D**), root fresh weight (**E**). Each bar represents mean $\pm$ SE (n = 3). Mean values marked with different letters are significantly different (p ≤ 0.05) by LSD test.

The gamma irradiation, seed moisture contents, and their interaction had a significant effect on maximum germination, seedling survival percentage, seedling length, and fresh weight of stem and root (Table 1). The highest and lowest seed germination percentages were observed in the interaction effects of 13% seed moisture with an irradiation dose of 400 Gy (98.89%) and 19% seed moisture with an irradiation dose of 100 Gy (82.22%), respectively (Fig. 1a).

**Table 1:** Analysis of variance of the effect of gamma irradiation and different seed moisture contents on some morphological, biochemical and molecular aspects of bread wheat.

PPO exp	PPO	POD exp	POD	CAT exp	CAT	RL	SL	RDW	SDW	RFW	SFW	SEL	SSP	Gmax	df	Source
0.02**	0.11**	0.44**	1.16**	69.34**	1.75**	4.87^ ns^	0.18^ ns^	0.003^ ns^	0.032^ ns^	6.23**	6.29**	251.67**	1415.29**	61.02**	2	Moisture
1.43**	0.25**	1.23**	0.94**	43.30**	4.67**	79.73**	83.31**	0.015**	0.016**	0.95**	0.85**	578.03**	1940.56**	55.77**	4	Gama
0.03**	0.15**	0.20**	0.16**	37.28**	1.31**	4.01^ ns^	11.18^ ns^	0.005^ ns^	0.004^ ns^	0.62**	0.27**	17.49**	670.50**	62.86**	8	Moisture $\times$ Gama
0.002	0.003	0.002	0.003	0.04	0.009	4.60	6.69	0.002	0.003	0.17	0.08	1.03	20.46	18.02	30	Error
16.67	7.54	13.12	8.67	10.85	8.52	30.33	21.69	33.90	25.66	33.35	17.93	3.53	7.50	4.61		CV

*Gmax* maximum germination percent, *SSP* seedling survival percent, *SEL* seedling length, *SFW* stem fresh weight, *RFW* root fresh weight, *SDW* stem dry weight, *RDW* root dry weight, *SL* stem length, *RL* root length, *CAT* catalase activity, *CAT exp* relative expression of catalase gene, *POD* peroxidase activity, *POD exp* relative expression of peroxidase gene, *PPO* polyphenol oxidase activity, *PPO exp* relative expression of polyphenol oxidase gene, *ns* non-significant.

**,*Significant at 1% and 5% levels of probability, respectively.

The results showed that with increasing the dose of gamma irradiation at different contents of seed moisture the percentage of survival and seedling length decreased (Fig. 1b,c). Seedlings showed the highest and lowest survival percentage in the interaction of 19% seed moisture with an irradiation dose of 100 Gy and the in 19% seed moisture with a dose of 400 Gy, respectively (Fig. 1b).

Combination treatments including 7% moisture and 100 Gy irradiation dose showed the highest fresh weight of stem and root (Fig. 1d,e). The regression equation showed that 234.21 and 297.8 Gy irradiation doses reduced 30% of the fresh weight of stem and root, respectively (Table 2).

**Table 2:** Proper equation for 30% growth dose reduction.

Traits	30% growth dose reduction	Equation	Coefficient of determination
Stem fresh weight	234.11	Y = − 0.000009X^2^ + 0.002174X + 1.754	0.927
Root fresh weight	297.89	Y = − 0.00189X + 1.66	0.90
Stem dry weight	321.32	Y = − 0.000001X^2^ + 0.000271X + 0.2385	0.93
Root dry weight	245.41	Y = − 0.000102X + 0.1987	0.96
Stem length	250.14	Y = − 0.000113X^2^ + 0.03030X + 12.84	0.90
Root length	221.16	Y = 0.00579X + 9.34	0.90

**Figure 2:**
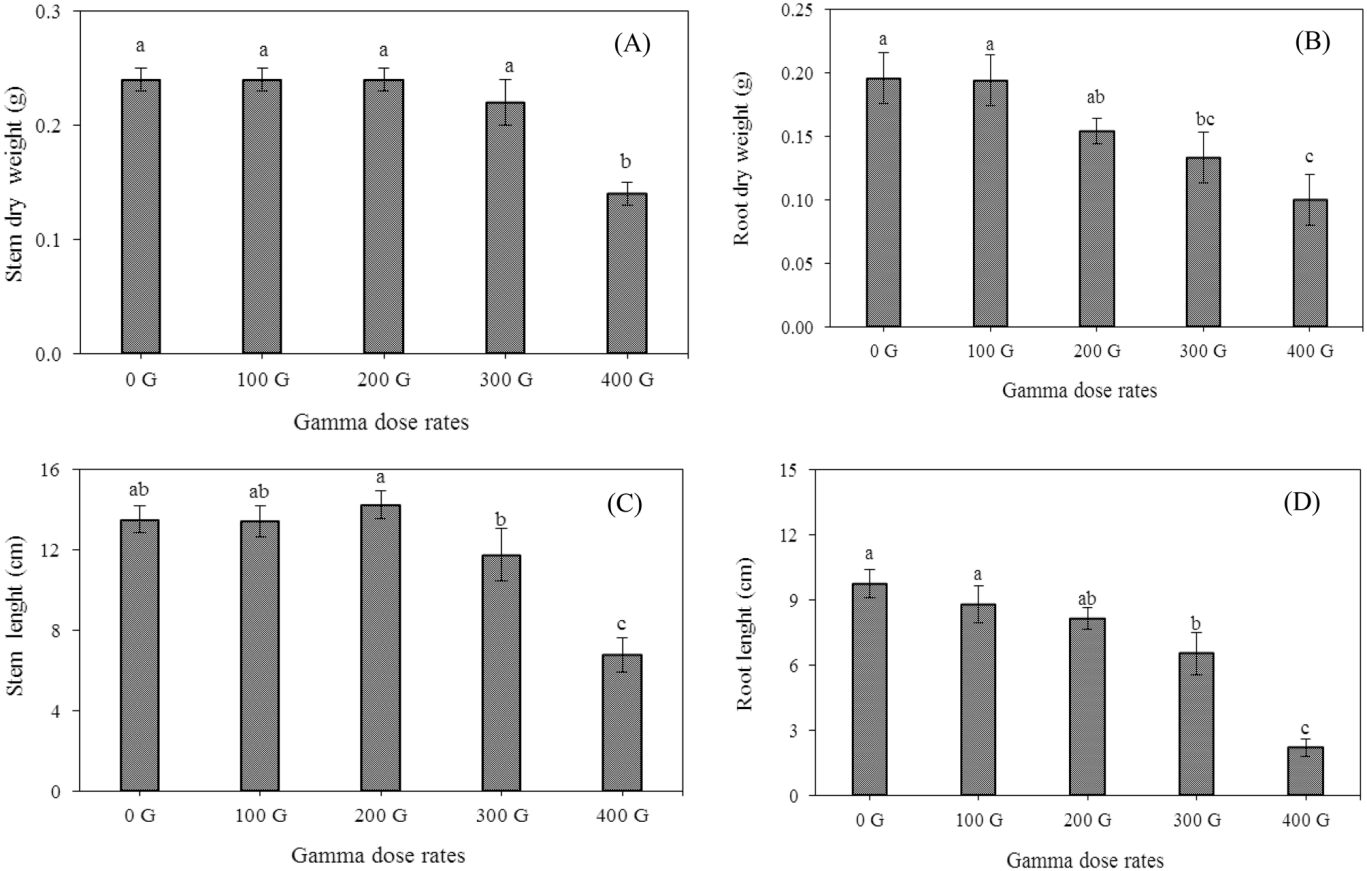
Effects of gamma dose rates on stem dry weight (**A**), root dry weight (**B**), stem length (**C**), root length (**D**). Each bar represents mean $\pm$ SE (n = 3). Mean values marked with different letters are significantly different (p ≤ 0.05) by LSD test.

Gamma irradiation doses had a significant effect on the stem and root dry weight, and stem and root length (Table 1). The dry weight of stem and root was reduced significantly in response to increasing gamma irradiation dose (Fig. 2a,b). The lowest stem dry weight was observed in gamma irradiation at 400 Gy, and the highest was observed at 100, 200, and 300 doses, which was not significantly different from the control (Fig. 2a). The regression equation showed that 321.32 and 245.41 Gy irradiation dose reduced 30% of stem and root dry weight, respectively (Table 2).

Stem and root length decreased significantly with increasing gamma irradiation dose (Fig. 2c,d). The maximum and minimum stem lengths were obtained by irradiation with doses of 200 and 400 Gy, respectively (Fig. 2c). The lowest root length was found in irradiation at a dose of 400 Gy, while its highest was observed in the control, which was not significantly different compared to the dose of 100 Gy (Fig. 2d). Regression equation showed that 250.14 and 221.16 Gy irradiation doses reduced 30% of Stem and root length, respectively (Table 2).

### Antioxidant enzyme activity

**Figure 3:**
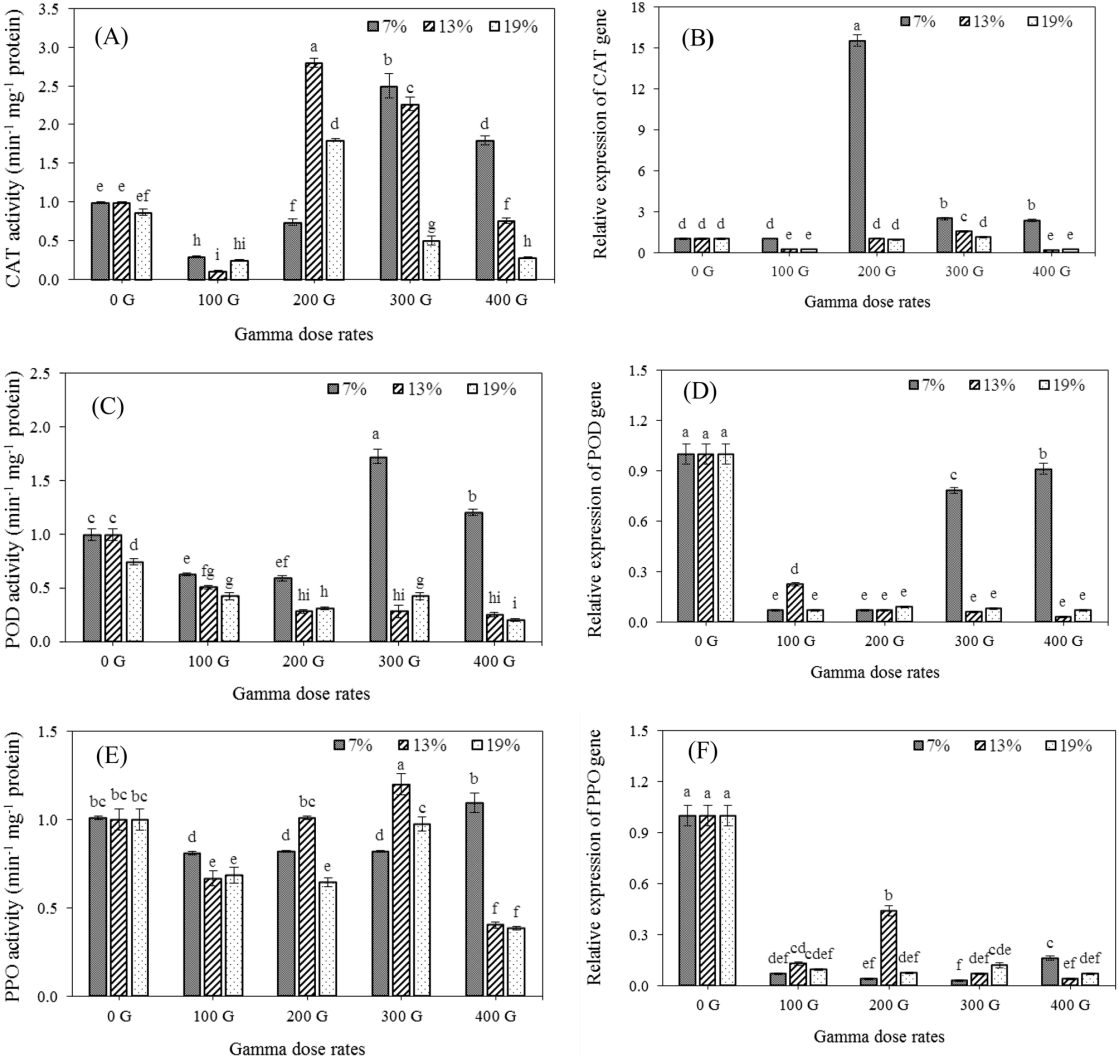
Effects of gamma dose rates and different seed moisture contents on catalase activity (**A**), relative expression of catalase gene (**B**), peroxidase activity (**C**), relative expression of peroxidase gene (**D**), polyphenol oxidase activity (**E**), relative expression of polyphenol oxidase gene (**F**). Each bar represents mean $\pm$ SE (n = 3). Mean values marked with different letters are significantly different (p ≤ 0.05) by LSD test.

The antioxidant enzyme activity such as CAT, POD, and PPO was significantly affected by gamma irradiation, seed moisture contents, and their interaction (Table 1). The CAT activity declined under 100 and 200 Gy irradiation doses at 7% seed moisture content but it increased in 300 and 400 Gy at this moisture level. The highest and lowest CAT activity in 13% and 19% seed moisture content was detected under 200 and 100 Gy irradiation doses, respectively (Fig. 3a).

The activity of POD reduced under all irradiation doses in 13% and 19% seed moisture content but increased in 300 and 400 Gy treatments in seeds containing 7% moisture. The highest activity of the POD enzyme was observed in the interaction of 300 Gy irradiation and 7% moisture content (Fig. 3c). The highest PPO activity in 13% and 19% seed moisture content was detected under 300 Gy irradiation dose, while it was shown in 7% seed moisture under 400 Gy irradiation (Fig. 3e).

### Relative expression of antioxidant enzyme gene

The relative expression of the antioxidant enzyme gene was significantly affected by gamma irradiation, seed moisture contents, and their interaction (Table 1). During the post-irradiation period, the expression of genes encoding antioxidant enzymes varied in seedlings with varying moisture contents. The highest level of relative expression of CAT was observed in seedlings with 13% and 19% moisture under gamma irradiation at a dose of 300 Gy. While, in 7% seed moisture content, the expression of CAT was strongly enhanced by 200 Gy of gamma irradiation (Fig. 3b). The transcript level of peroxidase and polyphenol oxidase was significantly inhibited by all induced gamma irradiation doses in all moisture content (Fig. 3d,f).

## Discussion

Normally, after seed irradiation with various doses cannot be seen significant differences between the doses of the final germination percentage but delay in germination occurs greater with increasing irradiation dose^26,28^. However, in this study different germination percentage was observed in different treatments. Given the significant interaction between gamma irradiation dose and seed moisture content on the germination percentage of wheat seeds, this could be a reason for the difference in yield. Improving germination traits in the seeds of plants exposed to low doses of gamma radiation can be attributed to the effects of radiation on genes controlling these traits, stimulating hormones, activating enzymes involved in germination processes, and accelerating DNA repair. In addition, low doses of gamma radiation may help improve plant germination by accelerating cell division in meristematic tissues^29^. In accordance with our study, it was recorded that the germination and survival of *Cuminum cyminum* seedlings improved at low doses (100 Gy) but decreased at high doses (500 Gy)^30^. Hussain et al.^31^ reported a dose range of 0.5–5 kR as an effective dose to increase sunflower germination. Beyaz et al.^10^ reported that irradiation doses of 100 and 150 Gy had a stimulating effect on the germination of *Lathyrus chrysanthus*. In another study, improvement of germination percentage and germination index of *Lactuca sativa* was reported using gamma at 30 Gy^13^. Aref et al.^32^ stated that 5 Gy had a stimulating effect on the germination of *Datura innoxia* but doses greater than 5 Gy had a debilitating effect on germination.

In this study, the percentage of survival and seedling length decreased with increasing gamma irradiation dose. Chromosomal change has been identified as a significant influence in limiting plant growth and survival^33^. An increase in the adverse effects of gamma-ray doses with increasing moisture may be due to a decrease in the production of ROSs by a decrease in seed moisture. Similar to the results of this study, Majeed et al.^34^ reported a significant reduction in growth traits and yield of chickpeas in gamma radiation at doses of 0.8–3.6 kGy.

In general, ionizing irradiation through the production of free radicals may have adverse effects on traits such as germination, growth, and plant yield^35^. In addition, high doses of gamma irradiation through stress signals have adverse effects on the physiological and biochemical traits of plants^12^. Reduced seedling growth could be due to the effects of irradiation on the signaling pathway of growth regulatory factors such as cytokinins and at the highest doses growth reduction caused by cell oxidation^36^. However, the highest doses result in the maintenance of cell division in the G2 phase and have devastating effects on the genome^37^. However, exposure to low levels of ionizers has a beneficial effect on plant growth, which is called hormesis^38^. In this study, low levels of gamma doses (200–300 Gy) reduced growth by 30%, which is a desirable trait for wheat.

Water radiolysis causes the production of ROS due to the indirect and/or semi-direct effects of irradiation. Water radiolysis is the ionization and stimulation of a water molecule to remove electrons from the water molecule and produce H_2_O^+^ and e^−^. Therefore, irradiation of seeds with high moisture content can lead to the production of high levels of ROS. Therefore, in 13% and 19% moisture content, because of the abundance of water, ROS was produced in lower irradiation doses compared to 7% moisture content. This is why CAT activity under gamma irradiation increases by 200 Gy at 13% and 19% seed moisture and by 300 Gy at 7% seed moisture. Increased activity of CAT indicates that the ability to scavenge for singlet oxygen and H_2_O_2_ in wheat was improved by irradiation to reduce free radical-mediated damage. This improvement is dependent on the water content of cells. The greater amount of water required much less irradiation dose to produce ROS.

When plants are exposed to gamma irradiation, the ROS increases with increasing dose absorbed^13^. Clearing of cells from reactive oxygen species is a common task in living cells through enzymatic and non-enzymatic pathways^39^. Some studies have provided evidence of enhanced antioxidant enzyme activities after gamma irradiation^40,41^. Increased enzyme activity is primarily responsible for the plant's improved antioxidant capacity and activity following irradiation^42^. Catalase, peroxidase, and polyphenol oxidase are important enzymes in clear cells from reactive oxygen species.

The results of gene expression were almost confirmed by a biochemical investigation of these two enzymes. Antioxidant response to wheat irradiation may be reflected in gene expression as well. Antioxidant enzyme activity alterations may be traced back to changes in gene expression that occur after irradiation. This research could provide light on the molecular changes that occur in plants after exposure to irradiation. Prior investigators found a decrease in catalase activity when exposed to UV-B light^43–45^. To protect plants from free radicals and H_2_O_2_, catalase is the most effective antioxidant enzyme^46^. Reduced catalase activity may be caused by the peroxisome being destroyed by excessive lipid peroxidation^47^. Also, there are some different isoforms of CAT in wheat. In the present study, we used CAT3 primers and it would be expected that the others isoforms of CAT have more important roles to scavenge free radicals. The antioxidant defense system's peroxidase activity was crucial in scavenging H_2_O_2_. Polyphenol oxidase is also responsible for the oxidation of phenolic compounds^48^. Several investigations have demonstrated that UV-B irradiation enhanced polyphenol oxidase^23,24,45^. Reactive oxygen species cause indirect oxidative damage, although the amount of damage they cause varies with irradiation type due to differences in linear energy transfer. In addition, for these two enzymes, there are some different isoforms too which can be activated under oxidative stress more than studied isoforms to clean cells from ROS.

## Conclusion

In general, high levels of free radicals can lead to the overproduction of ROS in plants, eventually leading to oxidative stress. The increase in free radical content by increasing irradiation dose may be associated with reduced plant growth. Increasing gamma irradiation doses were reduced root and stem fresh and dry weight, and root and stem length. Morphological analysis showed that the dose of 200–300 Gy reduced seedling growth by 30%, which is suitable for wheat cultivars. Some of the benefits of reducing vegetative growth in wheat are resistance to stem lodging, reduced vegetative growth duration, and less labor intensity at harvest. Also, 200–300 Gy at different moisture contents in molecular and biochemical studies was appropriate for the expression of defense enzymes. Due to the phenomenon of cross-tolerance, the results of this study can provide a useful basis for further research on genetic diversity in order to the selection of wheat cultivars resistant to other biotic and abiotic stresses.

## Materials and methods

### Seed moisture content treatment

Wheat seeds (*Triticum aestivum* L. var. Mahdavi) were adjusted to 7, 13, and 19% moisture contents. For this purpose, the seeds were hydrated by direct addition of some distilled water, then dried on silica gel (4:1 to seed) in a closed container at different intervals. The moisture content was confirmed using a moisture meter (Model PM-600, Japan).

### Seed irradiation treatment

After the confirmation of moisture contents, seeds were immediately irradiated at the following doses: 0, 100, 200, 300, and 400 Gray at the Nuclear Science and Technology Research Institute in Karaj, Iran. A dose rate of 0.864 kGy h^−1^ was used for gamma irradiation with the source of ^60^Co. They were used immediately to determine germination percentage, seedling growth, expression and activity of some antioxidant enzymes.

### Morphological traits

The germination test was carried out as a factorial experiment based on a completely randomized design with three replications using varying moisture contents and irradiation doses as two factors. Any replication included 30 healthy and uniform wheat seeds on 2 sheets of filter paper that were soaked with distilled water. In a growing room, seeds germinated under carefully monitored conditions (dark, mean temperature 25 °C, and relative humidity 80%). Germination was recorded on alternate days for 10 days and each seed with a radicle more than 2 mm was recorded as a germinated seed. Seedlings were weighed and measured after 10 days for length, fresh weight, and dry weight to determine whether or not they had germinated successfully. Maximum germination percent as a number of germination seeds/total number of seeds, stem and root fresh weight, stem and root dry weight, and stem and root length were measured. To measure organ length, plants were randomly selected from each replicate and stem length and root length were measured with a tape measure. For dry weight measurement, stems and roots were dried separately at 60 °C for 48 h and their weights were fixed.

In a greenhouse at the Gorgan University of Agricultural Sciences and Natural Resources, Iran, the effect of seed moisture content and gamma-ray doses on seedling growth were investigated under a completely randomized design (CRD) with three replications and ten wheat seedlings in each replication (pot). Seedling survival percent and seedling length were recorded on 14 days' plant. Sampling was done on four-leaf seedlings, and then was frozen in liquid nitrogen and stored at − 80 °C to be later used for antioxidant activities and the analysis of gene expression.

### Antioxidant enzyme activity

In order to obtain soluble protein, the samples were homogenized in 5 ml extraction buffer Tris–HCl, pH 7.8 with 10% (v/v) glycerol, and centrifuged for 15 min at 15,000 rpm and 4 °C. Bradford's method was used to determine the soluble protein concentration by using Bovine Serum Albumin (BSA) as a standard^49^.

H_2_O_2_ consumption was used to measure catalase activity (CAT; EC 1.11.1.6). The reaction mixture contained 3 ml phosphate buffer 0.5 M (pH 7.0), 5 µl of 30% (v/v) H_2_O_2_, and 50 µl of crude enzyme extract. The H_2_O_2_ decomposition was investigated according to the reduction in absorbance at 240 nm using a spectrophotometer (CE2021, Cecil, Cambridge, UK). Data expressed in ∆A min^−1^ mg^−1^ protein^50^.

The oxidation of guaiacol led to an increase in absorbance at 470 nm, which was used to determine peroxidase activity (POD; EC 1.11.1.7). The reaction mixture contained 3 ml potassium phosphate buffer 0.5 M (pH 7.0), 3 µl guaiacol 0.2 M, 10 µl of 30% H_2_O_2_, and 50 µl of crude enzyme extract The monitoring of absorbance was carried out at 470 nm using a spectrophotometer (CE2021, Cecil, Cambridge, UK). Data expressed in ∆A min^−1^ mg^−1^ protein^51^.

The oxidation of pyrogallol led to an increase in absorbance at 420 nm by a spectrophotometer (CE2021, Cecil, Cambridge, UK), which was used to evaluate polyphenol oxidase activity (PPO; EC 1.14.18.1). After mixing together 3 ml potassium phosphate buffer 0.5 M (pH 7.0), 50 µl of pyrogallol 0.2 M, and 50 µl of enzyme extract, the change of absorbance were recorded for two minutes. Data expressed in ∆A min^−1^ mg^−1^ protein^52^.

### Relative expression of antioxidant enzyme gene

Biozol Buffer was used to extracting total RNA from 100 mg wheat leaves (BioFlux, Japan). This study used a Nanophotometer (IPMLEN model p300) to determine the amount of RNA present, and samples were electrophoresed in an Ethidium Bromide-1.5% agarose gel. Genomic DNA samples were removed by DNase treatment using fermentase construction. DNase treatments were performed using 2 µg RNA, 1 U of DNaseI enzyme, 1 µl of DNaseI buffer, and 10 U of RNase inhibitor (Ribolock) (Thermo Scientific, USA) and DEPC water up to 9 µl for 30 min at 37 °C. For total RNA quality determination, the traditional method of gel electrophoresis was used. The integrity of total RNA was assessed on the basis of visualization of 28S and 18S ribosomal RNA subunits under gel documentation system 2000 (Bio-Rad, München, Germany).

Samples were then treated with a 25 mM EDTA buffer and incubated for 10 min at 65 °C. Each sample's 5 µg total RNA was used to synthesize the first strand of cDNA, along with 0.5 µg Oligo (dT) primer and DEPC water up to 11 µl for 5 min at 70 °C. As soon as this was done, the reaction mixture was adjusted to 19 µl using DEPC-treated water and incubated at 37 °C for 5 min with the additions of 4 µl from the 5 × cDNA reaction buffer, 2 µl of dNTP (10 mM dNTP mix), and 20 U of Ribolok RNase inhibitor enzyme. By incubating each sample with 200 U of Revert Aid enzyme (MMLV-RT) for 60 min at 42 °C, the reactions were eventually stopped for 10 min at 70 °C.

Primers for studied genes (CAT, POD, and PPO) as well as Glyceraldehyde-3-Phosphate Dehydrogenase (*GAPDH*) as reference genes were created with the help of Primer3 online software^53^ according to 3′-UTR region for all sequences. Table 3 lists the primer properties and accession numbers that were used during primer design.

**Table 3:** Primer sequences used in quantitative real time PCR.

Primer name	Sequence	Product Size	Annealing temperature	Accession number
**CAT**				
For	3-AAGGAGGAGGGAGGCAGTC-5̒	106	60.74	AF475100
Rev	3-CAAGGCTACACGCACACAAC-5		60.37	
**POD**				
For	3-GACCAGGACCTCTTCACCAA-5̒	224	60.09	AB518867
Rev	3-ACGATGGTCTGCACAAAGG-5̒		59.69	
**PPO**				
For	3-GCGACACCAGCTTCGTCTTC-5̒	227	63.42	AY596267
Rev	3-GTACTCCTTCCGGCCCTTCTT-5		62.98	
**GAPDH**				
For	3-CGGAAAGTTGACTGGAATGG-5̒	202	60.49	EF592180
Rev	3-GGACCTGTTGTCACCCTGAA-5̒		60.97	

*CAT* catalase, *POD* peroxidase, *PPO* polyphenol oxidase, *GAPDH* glyceraldeyde-3-phosphate dehydrogenase.

cDNA synthesized quality was surveyed by housekeeping gene *GAPDH* in standard PCR under the following conditions: 3 min at 95 °C for 1 cycle, 10 s at 95 °C and 10 s at 62 °C and 20 s at 72 °C for 35 cycles, 5 min at 72 °C for 1 cycle. Specific gene was surveyed after verification of cDNA synthesized in standard PCR.

A quantitative real-time PCR was performed using an iCycler thermal cycler (Bio-Rad, Hercules, CA) with a reaction volume containing 5 µl of diluted cDNA, 10 µl of 2X SYBR Bio Pars PCR Master Mix, and 1 µl of each gene-specific primer (10 pmol) in a final volume 20 µl with double distilled water. PCRs, keep in mind that the following conditions must be met: 3 min at 95 °C for 1 cycle; 10 s at 95 °C, 10 s at 62 °C and 10 s at 72 °C for 35 cycles, and 2 min at 72 °C for 1 cycle. The specificity of amplicons was verified by melting curve analysis (55–95 °C) after 81 cycles. Two biological and three technical replicas of each sample were used for real-time PCR analysis. Relative expression was computed using the 2^−∆∆T^ method.

### Data analysis

The results from the seed morphological and biochemical traits data were analyzed using SAS 9.2 and the least significant difference (LSD) test was employed to compare treatment means. The gene expression level that was analyzed using GenEx software (version 6.0) was calculated using Ct (Comparative CT (2^−ΔΔCT^) comparison method.

### Ethical approval

We confirm that all the experimental research and field studies on plants (either cultivated or wild), including the collection of plant material, complied with relevant institutional, national, and international guidelines and legislation. All of the material is owned by the authors and/or no permissions are required.

## Abbreviations

ROS: Reactive oxygen species
CAT: Catalase
POD: Peroxidase
PPO: Polyphenol oxidase
CRD: Completely randomized design
BSA: Bovine serum albumin
GAPDH: Glyceraldehyde-3-phosphate dehydrogenase
LSD: Least significant difference
